# Aligning Licensure and Recommended Use to Expedite Vaccine Access in Response to the Ongoing Bundibugyo Ebolavirus Epidemic in Central Africa

**DOI:** 10.3390/v18090957

**Published:** 2026-09-01

**Authors:** George L. O’Hara, Sam Halabi

**Affiliations:** 1 O’Neill Institute for National and Global Health Law, Georgetown University Law Center, Washington, DC 20001, USA; sam.halabi@georgetown.edu; 2 Department of Health Management & Policy, School of Health, Georgetown University Medical Center, Washington, DC 20057, USA

**Keywords:** Bundibugyo ebolavirus, Ebola virus, vaccination, licensure, governance

## Abstract

The ongoing *Orthoebolavirus bundibugyoense*—Bundibugyo virus (BDBV)—epidemic is the worst Ebolavirus outbreak in a decade and is rapidly expanding, with >4600 suspected cases in the Democratic Republic of Congo (DRC) alone. It has caused 2186 deaths and spread to neighboring countries. As BDBV has no approved vaccine, timely response will require adapted use of existing vaccines approved for other ebolaviruses and expedited development of novel vaccines targeted toward BDBV. Access to vaccines is fundamental to public health emergency response and generally depends on two expert bodies in each country: a national regulatory authority (e.g., the US Food and Drug Administration (FDA)) and a national immunization advisor. Receding traditional global health assistance compounds already-limited licensure and recommended use in the DRC and other low-income countries confronting recent outbreaks of Ebola, Marburg, and mpox, exacerbating access gaps for high-risk populations. This commentary thus clarifies the critical importance of a robust national immunization advisor, coordinated closely with expanded licensure, in expediting access to vaccines and other essential epidemic response tools. Using the regulatory responses of the Republic of Rwanda and DRC, it demonstrates that clearer delineation of roles between these bodies is critical for effective response, can help prioritize self-sufficiency and greater regional public health cooperation, and will thus enable swifter control of Bundibugyo.

## 1. Introduction

The ongoing *Orthoebolavirus bundibugyoense*—Bundibugyo virus (BDBV)—epidemic in the eastern Democratic Republic of the Congo (DRC) is the largest Ebola outbreak since 2016 and is rapidly expanding. As of August 12, a mere two months after the DRC’s health ministry declared the epidemic, there were >4600 suspected cases, with 2186 deaths in the DRC, as well as 20 confirmed cases and 2 deaths in neighboring Uganda [1]. Mortality for this infection is high, having reached 30 to 50 percent in two previous outbreaks [2].

Despite the need for expeditious vaccine access to curb BDBV, there is no approved vaccine against the virus. There are several candidate vaccines, many of which are still experimental, for use against BDBV that differ by the ‘vector’, i.e., molecular platform, on which they deliver the immunizing antigen, ranging from recombinant vesicular stomatitis virus (rVSV) to adenovirus serotype 26 (Ad26), modified vaccinia Ankara (MVA), and chimpanzee adenovirus (ChAdOx1). While data on the protection these candidates offer against BDBV is mostly limited to preclinical studies, a phase 1 clinical trial of ChAdOOx1, a novel vaccine, began in late July 2026, wherein favorable data will enable fast-tracking of phase 2 and 3 trials to conclude later this year [3,4]. Moreover, preclinical and clinical studies have found evidence of protection against BDBV provided by existing vaccines designed to combat other ebolaviruses, e.g., *Orthoebolavirus sudanense* (SUDV) and *Orthoebolavirus zairense* (EBOV). Vaccination of a small group of non-human primates with rVSV-SUDV and again with rVSV-EBOV 14 days later provided 100% protection against BDBV [5], while serum samples from [human] patients in West Africa vaccinated against EBOV with either rVSV-EBOV, Ad26.EBOV or MVA-BN Filo exhibited cross-reactive antibody responses against BDBV [6].

The heterogeneity in evidence regarding anti-BDBV candidacy across this array of existing Ebola virus vaccines—and novel ones—has understandably met with cautious regulation by international health governing bodies, such as the World Health Organization (WHO), keen to avoid inconsistent regulation in fulfilling its global mandate. While prudent, this multilateral caution is at odds with this national, unilateral crisis: the DRC is bearing nearly the entire BDBV disease burden. For example, the World Health Organization currently possesses a stockpile of the rVSV-ZEBOV vaccine, having approved it for ZEBOV, but has not released it for pooled procurement against Bundibugyo due to a lack of randomized clinical data on its effectiveness against BDBV [2]. Affected countries must, then, in the immediate term, draw on their own expert regulatory bodies’ capacity to evaluate and license candidate vaccines themselves—which would enable procurement by donation or bilateral purchase, both independently of WHO approval—and identify high-risk populations for priority implementation. The limited regulatory capacity in these countries, though, necessitates agile stewardship of resources.

The purpose of this commentary is to clarify the critical, yet under-discussed, importance of expert bodies’ roles in responding to this Ebola outbreak, namely, the national regulator and the national immunization advisor. Using the regulatory responses of the DRC and Rwanda to Marburg virus (MARV) and mpox, we illustrate that limitations in regulatory capacity do not preclude regulatory agility and, crucially, that clear delineation of roles between these bodies is critical for an effective public health response to BDBV.

A national regulatory authority (NRA) and a national immunization technical advisory group (NITAG) are two distinct, complementary bodies that work together in a country’s public health system to manage and oversee vaccines. Crucially, this commentary argues that a more robust national immunization advisor that is more coordinated with the national regulator can both promote health sovereignty and improve responses to BDBV—and other epidemics—within and beyond these emerging agreements.

## 2. Increased Regulatory Agility in Coordination with Robust Usage Recommendations Expedited Access to Medical Countermeasures

First, NRAs can and should use a different evidentiary standard for vaccine and other medical countermeasure (MCM) approval to make a greater number of effective and safe MCMs available in response to urgent public need [7,8,9]. The NRA may then increase the availability of MCMs to these populations without making explicit recommendations regarding use. This is not a regulatory backdoor. A large proportion of MCMs that are granted accelerated approval are not given full approval due to the emergence of superior clinical alternatives and less-than-satisfactory confirmatory data [10].

Separately, NITAGs—which are best equipped to make explicit usage recommendations by iteratively monitoring clinical effectiveness—should more actively take the initiative in advising the public on the use of NRA-approved MCMs.

This is precisely how Rwanda FDA (the country’s NRA) addressed its recent *Orthomarburgvirus marburgense* (MARV) outbreak. It authorized administration of MARV vaccine doses within ten days of the MARV outbreak. It approved the importation of the cAd3-Marburg vaccine on September 30, 2024, three days after the government declared the outbreak.

As the vaccine was still in Phase 2 trials, Rwanda FDA judged that importation for use in a clinical trial inside Rwanda was the most robust justification for approval. Rwanda FDA drew on encouraging Phase 1 safety data and communicated closely with the vaccine’s developer and trial managers to approve a ring trial protocol. Subsequently, in consultation with national epidemiological surveillance, Rwanda FDA recognized that all contacts of initial cases were concentrated in a single overlapping ring, which would complicate effective randomization between immediate and delayed vaccination, and that ~70% were healthcare workers, a fact that more clearly necessitated immediate vaccination of this high-risk group and their contacts. Rwanda FDA thus adapted the trial format into an open-label single-arm phase 2 trial under which healthcare workers, miners and other high-risk groups would receive immediate vaccination [11]. An estimated 1700 individuals received the vaccine [11].

The Rwandan government declared that the epidemic was over on December 20, day 84, after six weeks without any new cases, with 66 cases and 15 deaths in total [11]. By accepting the risk of introducing a vaccine still under investigation but with promising safety data and prioritizing recommended use in high-risk populations, Rwanda accelerated access and swiftly curbed the MARV epidemic.

The integration of manufacturer vaccine safety data collected abroad with NITAG surveillance data in Rwanda proved agile in terms of making doses available via a domestic clinical trial. This approach can empower NRAs now confronting BDBV to design, execute and monitor emergency clinical trials of existing vaccines (e.g., Ervebo), emerging rVSV-based BDBV-specific vaccines, and other emerging BDBV vaccine vectors in high-risk populations [12]. This clinical-trial-based access approach holds promise in several African countries affected by or proximal to BDBV, as forty-five clinical research units across sixteen countries in the region were reported to have adequate capacity to conduct phase 1 clinical trials in a summary published by the Coalition for Epidemic Preparedness Innovations (CEPI) last month [13]. For the DRC, which, as of yet, lacks the capacity for Phase 1 trials, the application of an accelerated licensure pathway such as emergency-use authorization, which uses a modified evidentiary standard given an urgent public need, can prove similarly agile in expediting access to reasonably safe and effective vaccines.

To be clear, these measures for issuing more agile responses to BDBV do not and should not compromise regulatory standards. Put plainly, they are intended to meet the current requirements and do not preclude subsequent vetting of vaccines for full approval.

## 3. Sharper Division of Governance Between Licensure and Recommended Use Can Reduce Duplication of Efforts and Reliance on World Health Organization Prequalification, Particularly Where Regulatory Capacity Is Limited

Rwanda regulated the MARV vaccine more adroitly in part due to its adequate regulatory capacity, i.e., the technical expertise of regulators, with WHO Maturity Level 3 [14]. But it also leveraged the roles of its expert bodies. This is in contrast with the actions of the Congolese Pharmaceutical Regulatory Authority (ACOREP), the DRC’s NRA, and of the DRC’s NITAG, the Technical Consulting Group for Vaccines (French: Groupe Technique Consultatif pour la Vaccination (GTCV)), when the clade 1b mpox epidemic began in late 2023.

At the outset of the clade 1b epidemic, there were two frontline vaccines indicated for mpox: Modified Vaccinia Ankara (MVA-BN), fully approved by the U.S. FDA in 2019, and Lister Clone 16 strain m8 (LC16m8), fully approved by Japan’s NRA in 2022 [15,16]. Despite the approval of MVA-BN and LC16 by these NRAs and steeply rising case counts in late 2023 to over 14,000 for the year (which can be compared to ~5000 in 2022), ACOREP did not grant emergency approval of either until May and June 2024, respectively [15].

This delay precluded mpox vaccine donation for half a year. It also undermined the recommendation by the GTCV in February 2024 to administer MVA-BN and LC16 to adults and children at high risk of infection [17]. Post-approval, ACOREP spent months in donation negotiations disputing the terms of licensure, including indemnification for vaccine-related adverse events [18].

ACOREP’s caution was predictable given the previous trialing of MCMs in low-income African countries by developers from high-income countries. Such developers often do not ensure care for individuals in the host country who suffer adverse reactions. They have also, on occasion, excluded local vaccine scientists as peers in clinical research and declined to address long-term equitable access to MCMs resulting from these efforts. These historical lessons warrant national governments’ firm assertion of their sovereign right to regulatory workflow self-governance. Robust clinical data and evidence-based medicine, however, transcend borders: pediatric safety data, by mid-2024, showed virtually no serious adverse events for either vaccine. Moreover, by this time, several NRAs and NITAGs worldwide, most more stringent and experienced than ACOREP and GTCV, respectively, had approved both vaccines for pediatric use [16]. By refusing to assume liability in regard to this high-risk population despite these highly favorable risk profiles and approval by more sophisticated regulators, ACOREP imposed a case-by-case clinical medicine approach on population-level usage despite the evident incongruity with the public health need—all upstream of its own NITAG, the GTCV, which was set up expressly to monitor population health data.

Regulatory rigidity not only delayed supply by donation: it rendered the DRC even more dependent on external licensure as it sought supply by another route—international pooled procurement of vaccines granted “prequalification” (PQ) status by the WHO [19]. This avenue was protracted. The WHO did not grant PQ status to MVA-BN until September 2024 even though it had been receiving safety and effectiveness data from its manufacturer since 2023 [20]. Vaccination began in October 2024, ~1 year into the outbreak, and even then lab-based testing shortages prevented timely follow-up [18]. Regulation-induced delays in the DRC thus clearly undermined epidemic control. These delays in the DRC were exacerbated by other structural limitations shared by many low-income countries, such as political instability and limited cold-chain capacity, i.e., the ability to store vaccines at required low temperatures to avoid degradation [21].

ACOREP thus allowed concerns about liability, however understandable, to prescribe usage, effectively duplicating the function of internationally developed usage recommendations, and those of the GTCV, for mpox vaccines. This duplication stymied vaccine access for key populations when manufacturers offered doses. The relationship between licensure and recommended use during emergencies can grow more effective with a more clearly defined interface between their respective purviews. To build these capabilities, newer NITAGs have increased NRA-NITAG information exchange and integrated NRA experts as ex officio NITAG members [22].

These measures for more transparent division of governance would allow the NRA in a country affected by BDBV to delegate post-approval monitoring, and, with it, concerns about vaccine tolerance, completely to the NITAG. The NRA, in turn, could focus its regulatory capacity, which is already limited (i.e., WHO Maturity Level 1–2, e.g., ACOREP in the DRC), entirely on licensure, i.e., the approval process itself. Finally, an NRA-NITAG relationship with minimal duplication would better enable each body to seek specialized regulatory guidance from more stringent regional international counterparts. If the NRA lacks sufficient capacity to grant emergency approval on an expedited timeframe, for example, then it may refer to the judgment of a leading regional NRA (i.e., ML 3+) as a form of ‘deemed approval’ to inform its own authorization decision [23].

## 4. Limitations, Challenges and Solutions

There are notable challenges and caveats to more clearly divided licensure and recommended use in response to BDBV and otherwise. First, limited health ministry funding for NITAGs poses a significant challenge to fortifying NITAGs’ responses to BDBV. NITAGs in Sub-Saharan Africa and elsewhere cannot afford to employ dedicated staff. Most NITAG core members occupy separate primary clinical or health ministry positions [24]. Moreover, more pro-active usage recommendations, as advocated here, are more likely mistrusted and ignored by clinicians and the public if delivered without clear communication of their evidence bases [25].

Second, the purviews of NRAs and NITAGs must overlap in specific instances to respond to outbreaks. Supply adequacy depends on NRA authorization for importation in tandem with NITAG biosurveillance for dose allocation. ACOREP and GTCV may coordinate these tenets of supply with minimal duplication by designating one representative to form a two-person team tasked with conveying regulatory hurdles and epidemiologic data to CEPI, which is expediting BDBV vaccine development, and GAVI, which oversees the global emergency stockpile of Ervebo vaccines [26]. Measured personnel deployment as such can effect swifter vaccine importation and dose allocation, particularly as Ervebo is now poised for emergency introduction into the DRC to combat BDBV following the order given by the Africa CDC director earlier this month [27].

Post-approval monitoring also requires considerable overlap between NRAs and NITAGs. NRAs manage concerns about liability and indemnification due to vaccine adverse events and thus may demand direct oversight of post-approval monitoring. NITAGs, meanwhile, possess the epidemiologic and medical specialty expertise (pediatrics, infectious disease, and immunology) needed to execute this monitoring.

Finally, differences in epidemiology, vaccine availability and prior experience between Marburg in Rwanda and mpox clade 1b in the DRC may affect the generalizability of governance lessons from the former to BDBV today. Marburg in Rwanda was confined to a few dozen healthcare workers and highly deadly. The highly centralized structure of the Rwandan government rapidly aligned political capital with the capacity of its FDA to fast-track vaccine authorization. Community-based medical insurance, ‘mutuelles de santé’, and contact-tracing capacity tested by prior HIV and malaria epidemics cover over 80 percent of the population and were thus well-poised to identify key populations and allocate doses [28,29]. Mpox clade 1b in the DRC, by contrast, had lower mortality among healthy non-pregnant adults and spread broadly before it was recognized as a novel mpox strain and afforded concordant concern. It was of secondary importance for a central government confronting far deadlier outbreaks of polio, cholera and Zaire ebolavirus across a population nearly eight times that of Rwanda. Lastly, Kinshasa exerts peripheral control, to put it generously, over much of the country’s interior and eastern mining and fishing towns where mpox and now BDBV have spread, spanning a landmass nearly ninety times that of Rwanda [30]. This national context impeded timely contact tracing and delivery of authorized vaccines.

## 5. Conclusions

This commentary demonstrates that robust and clearly defined roles for technical advisory in collaboration with licensure can be pivotal to guiding risk–benefit assessment to ensure swifter implementation of existing and emerging vaccines using rVSV, adenovirus, and MVA platforms in response to the current Ebola outbreak in Central Africa. Beyond the regulatory concerns presented here, the complex geopolitical climate of the DRC, lack of infrastructure and resources for responding to the current BDBV outbreak, and logistical challenges of conducting clinical vaccine trials are important additional barriers to implementing vaccines in a timely manner in public health emergencies. Nevertheless, this proposed division of governance between licensure and recommended use prioritizes self-sufficiency and lends itself to regional cooperation within public health specialty expertise.

## Data Availability

No new data were created or analyzed in this study. Data sharing is not applicable to this article.

## Abbreviations

The following abbreviations are used in this manuscript:

ACOREP	Congolese Pharmaceutical Regulatory Authority (French, original: Autorité Congolaise de Réglementation Pharmaceutique)
BDBV	Bundibugyo ebolavirus
cAd3	Chimpanzee adenovirus type 3
CEPI	Coalition for Epidemic Preparedness Innovations
DRC	Democratic Republic of Congo
EBOV	*Orthoebolavirus zairense*
FDA	Food and Drug Administration
GAVI	Global Alliance for Vaccines and Immunization
GTCV	Groupe Technique Consultatif pour la Vaccination
LC16	Lister Clone 16 strain [of the vaccinia virus]
MARV	*Orthomarburgvirus marburgense*
MCMs	Medical countermeasures
ML	Maturity level
MVA-BN	Modified Vaccinia Ankara–Bavarian Nordic
NITAG	National immunization technical advisory group
NRA	National regulatory authority
PQ	Prequalification
rVSV	Recombinant vesicular stomatitis virus
SUDV	*Orthoebolavirus sudanense*
US	United States of America
WHO	World Health Organization

## References

[1] World Health Organization (2026). Ebola Bundibugyo Virus Disease Outbreak Democratic Republic of the Congo|Uganda Weekly External Situation Report 14, Data as of 16 August 2026.

[2] World Health Organization (2026). Ebola Disease Caused by Bundibugyo Virus—Democratic Republic of the Congo.

[3] Geddes L. (2026). The World’s First Bundibugyo Ebola Vaccine Has Entered Human Trials: Here’s Why It Matters.

[4] Shenai D. (2026). First volunteer gets Ebola vaccine—Three months after the outbreak began. Nature.

[5] Mire C.E., Geisbert J.B., Marzi A., Agans K.N., Feldmann H., Geisbert T.W. (2013). Vesicular Stomatitis Virus-Based Vaccines Protect Nonhuman Primates against Bundibugyo ebolavirus. PLoS Neglected Trop. Dis..

[6] Lhomme E., Wiedemann A., Ayouba A., Ben-Farhat S., Thaurignac G., Roy C., Beavogui A.H., Doumbia S., Kieh M., Leigh B. (2026). Cross-Reactive Bundibugyo Antibody Responses after Receipt of Licensed Ebola Vaccines. N. Engl. J. Med..

[7] Roberts J.N., Gruber M.F. (2015). Regulatory considerations in the clinical development of vaccines indicated for use during pregnancy. Vaccine.

[8] World Health Organization (2016). Fractional Dose Yellow Fever Vaccine as a Dose-Sparing Option for Outbreak Response: WHO Secretariat Information Paper.

[9] World Health Organization (2016). Polio vaccines: WHO position paper—March, 2016. Wkly. Epidemiol. Rec..

[10] Franco P., Jain R., Rosenkrands-Lange E., Hey C., Koban M.U. (2023). Regulatory Pathways Supporting Expedited Drug Development and Approval in ICH Member Countries. Ther. Innov. Regul. Sci..

[11] Nsanzimana S., Bigirimana N., Hatchett R., Bailey S., Butera N., Butera Y., Cramer J.P., Finan A., Forkin C.M., Hacker A.M. (2025). How Rwanda mounted a research response with an investigational vaccine just ten days into a Marburg outbreak. npj Vaccines.

[12] Joi P. Bundibugyo, the Rare Virus Causing a Deadly New Ebola Outbreak, Has No Vaccine Yet. Here’s What We Know. https://www.gavi.org/vaccineswork/bundibugyo-rare-virus-causing-deadly-new-ebola-outbreak-drc-has-no-vaccine-yet.

[13] Coalitions for Epidemic Preparedness Innovations. African Phase I Clinical Trial Units: Request for Information (RFI) Summary. https://cepi-tr.tghn.org/priority-diseases/filoviruses/.

[14] World Health Organization (2024). Senegal and Rwanda Achieve WHO Maturity Level 3 in Medicines Regulation.

[15] Japan Ministry of Health, Labour and Welfare, Quarantine Station. Mpox—Democratic Republic of the Congo (Translated from Japanese). https://www.forth.go.jp/topics/2024/20240719_00001.html.

[16] Grabenstein J.D., Hacker A. (2024). Vaccines against mpox: MVA-BN and LC16m8. Expert Rev. Vaccines.

[17] Mbala-Kingebeni P., Rimoin A.W., Kacita C., Liesenborghs L., Nachega J.B., Kindrachuk J. (2024). The time is now (again) for mpox containment and elimination in Democratic Republic of the Congo. PLoS Glob. Public Health.

[18] Cohen J., Bashizi A. (2024). ‘I’m Really Shocked.’ Children Not Being Vaccinated for Mpox in Congo. Science.

[19] World Health Organization (2022). Vaccines And Immunization for Monkeypox: Interim Guidance, 16 November 2022.

[20] World Health Organization (2024). WHO Prequalifies the First Vaccine Against Mpox.

[21] Guillaume D., Meyer D., Waheed D.-e.-N., Schlieff M., Muralidharan K., Chou V.B., Limaye R. (2023). Factors influencing the prioritization of vaccines by policymakers in low- and middle-income countries: A scoping review. Health Policy Plan..

[22] Chocarro L., Duclos P., Senouci K., Southern J. (2011). Consultation on interactions between National Regulatory Authorities and National Immunization Technical Advisory Groups. Expert Rev. Vaccines.

[23] Halabi S., O’Hara G.L. (2024). Preparing for the Next Pandemic—Expanding and Coordinating Global Regulatory Capacity. N. Engl. J. Med..

[24] Külper-Schiek W., Mosina L., Jacques-Carroll L.A., Falman A., Harder T., Kakarriqi E., Preza I., Badalyan A., Sahakyan G., Romanova O. (2025). Evaluation of National Immunization Technical Advisory Groups (NITAGs) of middle-income countries in the WHO European Region; a synopsis. Front. Public Health.

[25] Mello M.M., Walensky R.P., Osterholm M.T., Brennan T.A. (2026). Navigating Conflicting Vaccination Recommendations: Guidance for Clinicians. JAMA.

[26] Nishtar S. (2026). Financial innovation can deliver a Bundibugyo Ebola vaccine. Nat. Health.

[27] China Global Television Network (2026). Africa CDC Urges Urgent Vaccine Deployment as Ebola Outbreak Spreads Across DR Congo.

[28] Emeli I.M. (2024). The Rwandan Healthcare System: Can a Shifting Burden of Disease Threaten a Post-war Success Story?. Cureus.

[29] Julius N., John M., Emmanuel N. (2026). Public Health Achievements in Rwanda: A 21st Century Transformation. Public Health Chall..

[30] Karan A., Katz I.T., Jha A.K. (2018). Ebola in the DRC Is More About the DRC than It Is About Ebola. Health Aff..

